# Pilocarpine in Skin Diseases

**Published:** 1891-01-24

**Authors:** 


### PILOCARPINE IN SKIN DISEASES.

Pick, Klotz, Simon, and others holding Unna's views as to the physiology of the secretion of sweat recommend the use of pilocarpine in pachydermatous and erodermatous conditions, prurigo, pruritus senilis, icthyosis, exfoliative dermatitis, &c. They prefer small doses, $\frac{1}{8}$ or $\frac{1}{12}$ grain continued for a long time. We would, however, call attention to the fact that while no drug acts more certainly and accurately when given subcutaneously, given by the mouth doses even ten times larger may fail to cause appreciable perspiration. Indeed, we have heard of toxic effects occurring without the characteristic physiological effects.

